# Glucagon-like peptide-1 receptor agonists in psoriasis and psoriatic arthritis: emerging evidence and future research opportunities

**DOI:** 10.3389/fimmu.2026.1744308

**Published:** 2026-04-28

**Authors:** Giovanni Ciancio, Beatrice Maranini, Gilda Sandri, Gabriele Amati, Alessandra Bortoluzzi, Ettore Silvagni, Marcello Govoni, Dilia Giuggioli

**Affiliations:** 1 Chair of Rheumatology, University of Modena and Reggio Emilia, Modena, Italy; 2 Department of Medical Sciences, University of Ferrara, Ferrara, Italy; 3 Rheumatology Unit, Azienda Ospedaliera Universitaria Sant’Anna di Ferrara, Ferrara, Italy; 4 Rheumatology Unit, Azienda Ospedaliero-Universitaria Policlinico di Modena, Modena, Italy

**Keywords:** biologic therapy, early psoriatic arthritis, GLP-1 receptor agonists, inflammation, metabolic syndrome, psoriasis

## Abstract

**Background:**

Psoriasis (PsO) and psoriatic arthritis (PsA) are chronic immune-mediated diseases often associated with obesity, metabolic syndrome, and type 2 diabetes mellitus. Glucagon-like peptide-1 receptor agonists (GLP-1RAs), initially developed for T2DM, exert both metabolic and anti-inflammatory effects, which may offer therapeutic benefits for psoriatic disease, particularly in the early stages of PsA.

**Objectives:**

This review aims to evaluate the current evidence on GLP-1RAs in PsO and PsA, examine the underlying pathophysiological mechanisms, and highlight key areas for future research, with a particular emphasis on early PsA as a critical window for intervention.

**Methods:**

A narrative review in accordance with current methodological guidance was conducted on published studies concerning GLP-1RAs in PsO and PsA, including preclinical, clinical, and mechanistic research.

**Results:**

Several studies show that GLP-1RAs, particularly liraglutide and semaglutide, improve PsO severity, metabolic parameters, and inflammatory markers. These benefits extend beyond weight loss, suggesting a direct immunomodulatory effect. Two open-label trials in PsA patients with obesity indicated improvements in disease activity (MDA) alongside metabolic benefits. The trials, NCT06588296 (TOGETHER-PsA) and NCT06864026 (TOGETHER AMPLIFY-PsA), assessed ixekizumab with and without tirzepatide. However, evidence in PsA remains limited, with most studies constrained by small sample sizes and short follow-up periods. To date, no studies have specifically investigated GLP-1RAs in early PsA; however, early-stage disease may represent an optimal treatment window for maximizing therapeutic effects based on immunometabolic rationale.

**Conclusions:**

GLP-1RAs show promising effects in PsO and early-PsA may represent a potential treatment window for maximizing therapeutic effects based on immunometabolic rationale, although this concept requires validation in dedicated clinical studies.

## Highlights

- GLP-1RAs improve PsO severity and metabolic parameters other than weight loss in PsO.
- Evidence in PsA is preliminary but suggests potential benefits of GLP-1RAs in disease activity modulation.
- Ongoing trials are expected to clarify the role of GLP-1RAs in psoriatic disease and other immune-mediated inflammatory diseases.
- GLP-1RAs may represent a class effect, offering dual metabolic and immunomodulatory benefits with long-term impact on comorbidities.

## 1 Introduction

Psoriasis (PsO) is a chronic, immune-mediated inflammatory skin disorder affecting 2–3% of the global population (1). It is characterized by erythematous, scaly plaques most commonly involving elbows, knees, scalp, and trunk (2). Beyond its cutaneous manifestations, PsO is increasingly recognized as a systemic disease with a broad spectrum of associated comorbidities, including metabolic syndrome (MetS), cardiovascular disease, insulin resistance, and type 2 diabetes mellitus (T2DM) (3–5).

In parallel with this evolving disease concept, growing evidence points to a complex immunometabolic interplay linking dysregulated glucose metabolism, incretin biology, and chronic inflammation in PsO (6). Rather than representing a purely cutaneous condition, PsO appears to be a systemic metabolic–inflammatory disease in which insulin resistance, adipokine imbalance, and altered gut–endocrine signaling contribute to immune dysregulation long before the onset of clinically overt joint involvement (7).

Within this framework, incretins —particularly glucagon-like peptide-1 (GLP-1)— have emerged as key immunometabolic regulators rather than merely glucose-lowering hormones. GLP-1 exerts pleiotropic effects on innate and adaptive immune responses, endothelial function, and adipose tissue inflammation, thereby influencing multiple pathways relevant to psoriatic disease (8). Recent conceptual frameworks propose that metabolic stress and impaired incretin signaling may amplify interleukin (IL) 23/17–driven immune responses at the enthesis and synovium, thereby facilitating the transition from PsO to psoriatic arthritis (PsA) in genetically or immunologically predisposed individuals (9).

This immunometabolic crosstalk provides a biologically plausible link between metabolic dysfunction and musculoskeletal inflammation, and supports the hypothesis that targeting incretin pathways could interfere with disease evolution at a preclinical or early PsA. Consistent with this concept, up to 30% of individuals with PsO eventually develop PsA, a chronic inflammatory arthropathy affecting peripheral joints, entheses, and the axial skeleton (10). According to clinical phenotypes, PsA typically presents as asymmetric oligoarthritis, dactylitis, enthesitis, or spondylitis (11).

Early PsA may display nonspecific musculoskeletal symptoms, such as inflammatory arthralgia or morning stiffness, even in the absence of overt synovitis or radiographic erosions (12). This prodromal phase, defined as “early PsA” or “psoriatic musculoskeletal symptoms without arthritis”, represents a critical window of opportunity for disease interception, as defined by Zabotti et al. (12, 13). In this context, recent EULAR “Points to Consider” emphasize the importance of recognizing clinical and imaging features suggestive of progression from PsO to PsA, including arthralgia, enthesitis, subclinical synovitis on ultrasound or MRI, and elevated inflammatory markers (12).

At the pathogenic level, PsO and PsA share core inflammatory mechanisms driven by dysregulation of the IL17/IL23 and Tumour Necrosis Factor (TNF)-α cytokine axes (14), contributing to keratinocyte hyperproliferation, synovial inflammation, and enthesial disease (15–18).

These immune pathways are closely intertwined with metabolic abnormalities, particularly obesity and adipose tissue dysfunction. Visceral adiposity contributes to chronic low-grade inflammation through macrophage activation, altered T-cell responses, and adipokine imbalance (19–22). Notably, patients with PsA have a higher prevalence of MetS compared to those with PsO alone or rheumatoid arthritis (RA). Obesity is also associated with both increased PsA incidence and reduced therapeutic response to biologic agents (23, 24). In fact, visceral fat contributes to the expansion of Th17 cells and secretion of IL17 and IL23, which are the key drivers of psoriatic inflammation (24, 25). Moreover, γδ T cells, frequently found in psoriatic skin, have been identified as responsive to metabolic modulation (26).

Beyond obesity, insulin resistance and hyperinsulinemia appear to play an active role in sustaining inflammatory arthritis rather than representing mere epiphenomena of chronic inflammation. Insulin signaling directly influences immune cell metabolism, macrophage polarization, and T-cell differentiation, thereby shaping inflammatory responses within musculoskeletal tissues (27).

**Figure 1:**
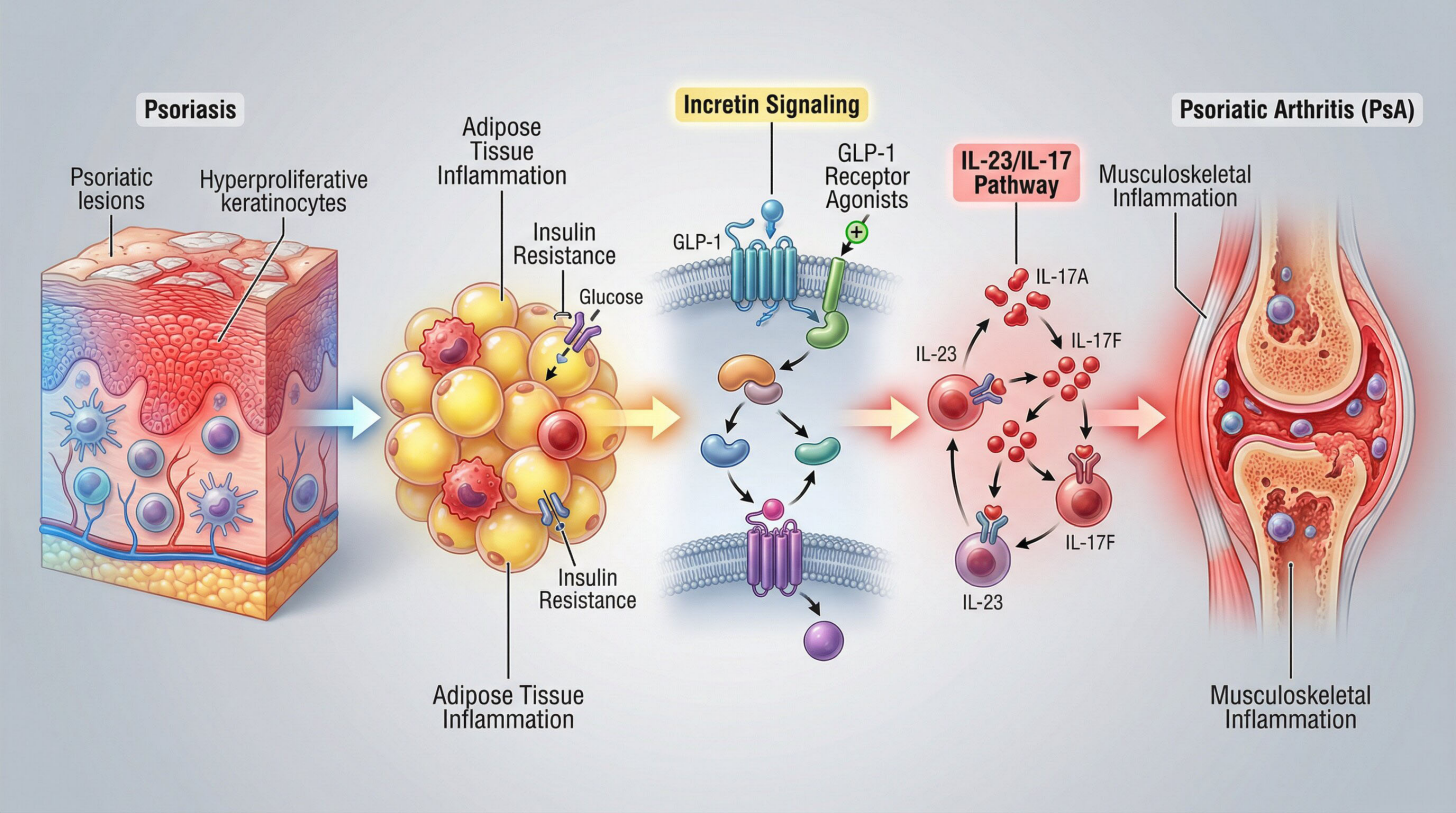
Schematic illustration of the immunometabolic continuum linking PsO to PsA, highlighting the roles of insulin resistance, adipose tissue inflammation, incretin signaling, and IL-23/IL-17–driven musculoskeletal inflammation, as well as the potential modulatory effects of GLP-1 receptor agonists.

Increasing evidence from immunometabolic research supports a bidirectional relationship between metabolic dysfunction and inflammatory arthritis, whereby chronic inflammation promotes insulin resistance, which in turn amplifies synovial and entheseal inflammation (28). In PsA, insulin resistance has been associated with higher disease activity, greater pain burden, and reduced therapeutic responsiveness, reinforcing the rationale for metabolic interventions as part of a disease-modifying strategy (29). As illustrated in **Figure 1**, insulin resistance, adipose tissue inflammation, and impaired incretin signaling converge at the skin–joint axis to amplify IL-23/IL-17–driven immune activation and disease progression.

In line with these observations, weight loss has emerged not only as a supportive strategy but also as a disease-modifying intervention in PsA (30). Both clinical trials and observational studies have shown that intentional weight loss, whether through lifestyle change, bariatric surgery, or pharmacotherapy, is associated with reduced disease activity, improved joint symptoms, and enhanced response to biologics (31, 32). Among pharmacologic options, glucagon-like peptide-1 receptor agonists (GLP-1RAs) have gained particular interest over recent years (33, 34). In addition to improving glycemic control, GLP-1RAs promote satiety, reduce visceral adiposity, and exert indirect anti-inflammatory effects (35). Experimental and clinical data indicate that GLP-1RAs reduce circulating pro-inflammatory cytokines, improve endothelial function, and favour macrophage polarization toward an anti-inflammatory M2 phenotype, thereby attenuating adipose tissue inflammation. These pleiotropic effects likely underlie their broader benefits in inflammation-driven comorbidities such as NAFLD/NASH and atherosclerosis (35, 36).

Preclinical studies further suggest that GLP-1RAs may modulate key cytokine pathways implicated in psoriatic disease, including NF-κB, IL-6, IL17, and IL23, while small clinical studies have reported improvements in skin disease severity in PsO (37). However, despite these promising findings, the potential role of GLP-1RAs in PsA, particularly as a dual-action agent targeting both metabolic and immune dysregulation, remains insufficiently explored.

This narrative review summarizes the current clinical evidence on GLP-1RAs in PsO and PsA and discusses future research directions aimed at evaluating their potential role in intercepting PsA development and improving outcomes in patients with complex immunometabolic profiles. The review was conducted in accordance with established methodological guidance for narrative reviews (38). Details of the literature search strategy and PRISMA flowchart are provided in the **Supplementary Materials**, and the main findings are summarized in **Table 1**.

**Table 1:** Clinical studies of GLP-1 receptor agonists in psoriasis (PsO) and psoriatic arthritis (PsA) (chronological order of publications).

Study	Year	Design	GLP-1RAs used	Population (patients)	Outcome	Weight/ MetS change	Notes
Drucker et al. (47)	2011	Commentary	GLP-1RAs (various)	N/A	N/A	N/A	First article to propose mechanistic link between GLP-1, PsO, and adipose-driven inflammation
Hogan et al. (39)	2011	Case-series with mechanistic *in vitro* work	Liraglutide	Two patients with PsO + T2DM (plus *in vitro* assays on iNKT cells)	↓PASI; ↑circulating iNKT cells and ↓plaque iNKT cells; GLP-1 dose−dependently inhibited cytokine secretion *in vitro*	N/A	Demonstrates direct immunomodulatory effect on iNKT cells; cytokine inhibition but no effect on degranulation; supports immune−mediated mechanism rather than metabolic only
Ahern et al. (40)	2012	Prospective cohort	Liraglutide	7 PsO + Obesity + T2DM	↓ PASI, increases circulating iNKT cell number and modulates monocyte cytokine secretion	↓ BMI	Clinical improvement may reflect combo of weight improvements and direct immunomodulation
Reid et al. (52)	2013	Case report (N = 1)	Liraglutide	54−year−old man, PsO + obese	↓ PASI	↓ BMI	First report combining liraglutide with acitretin in PsO without T2DM.
Al Badri et al. (41)	2014	Narrative review	GLP-1RAs (various)	PsO + MetS or T2DM	Anecdotal improvements in clinical and histopathologic psoriasis severity	↓ BMI and glycemia	First mechanistic review proposing GLP-1 immune-modulating role in PsO
Buysschaert et al. (26)	2014	Prospective cohort	Exenatide/Liraglutide	7 PsO + T2DM	↓ PASI; ↓in epidermal thickness (histological); ↓ γδ T cells	↓ BMI and glycemia	First study linking clinical improvement to T-cell reduction
Faurschou et al. (50)	2014	Case report (N = 1)	Liraglutide	59-year-old man, PsO + T2DM	Marked improvement in PsO symptoms (itching, scaling) within days	↓ BMI and glycemia	PsO improved before significant weight loss; suggests possible anti-inflammatory effect
Faurschou et al. (59)	2015	Open-label trial	Liraglutide (1.2-1.8 mg/day)	PsO + Obesity	↓ PASI (no significant)	↓ BMI and cholesterol levels	Clear dose–response relationship
Xu et al. (42)	2019	Prospective cohort study	Liraglutide (1.8 mg/day)	7 PsO + T2DM	↓PASI; histologic improvement of skin lesions	↓ BMI, waist circumference, ↓ HOMA-IR, ↓ HbA1c	Improvement in PASI and metabolic markers
Yang et al. (51)	2019	*In vitro* study	Liraglutide	Human keratinocytes (cell culture)	GLP-1RA inhibits inflammatory pathways in keratinocytes	No weight/MetS data (*in vitro*)	Liraglutide activated AMPK, impairing inflammatory signals in keratinocytes, suggesting potential anti-inflammatory properties
Chen et al. (56)	2021	Animal model	Liraglutide	Obese diabetic mice with psoriasiform skin	↓ PASI-like lesions; ↓ IL17, IL23IL23, IL22	N/A	Indicates that liraglutide suppressed pro-inflammatory cytokines through IL−23/Th−17 pathway in obese diabetic psoriasiform model
Costanzo et al. (57)	2021	Case report	Semaglutide	73-years-old man, PsO + obese + T2DM	↓PASI	↓ BMI and glycemia	Severe PsO previously unresponsive to biologic therapy; semaglutide led to dramatic clearing alongside metabolic improvements
Chang et al. (43)	2022	Systematic review & meta-analysis	Liraglutide	PsO + T2DM	↓ PASI	↓ BMI, HbA1c (no significant)	Supports direct anti−inflammatory/modulatory action of liraglutide beyond metabolic change
Lin et al (55).	2022	Randomized controlled trial (open-label)	Liraglutide (1.8 mg/day)	25 PsO + T2DM; liraglutide group n = 12, control (conventional therapy) n = 13	↓PASI greater in liraglutide group *vs* control; skin histopathology improved; ↓IL−17, IL−23, TNFα expression in skin *vs* control	In intervention group ↓ BMI *vs* control; ↓ waist circumference	Supports anti-inflammatory mechanism *via* downregulation of IL−17/IL−23/TNFα
Malavazos et al. (58)	2023	Prospective study	Semaglutide	1 PsO + Abdominal obesity + T2DM	↓ PASI + ↓ epicardial inflammation (*via* coronary computed tomography angiography)	↓ Visceral adiposity	First study showing GLP-1RA reduces fat-associated inflammation (imaging evidence)
Matwiejuk et al. (44)	2022	Narrative review with case observations	GLP-1RAs (various)	PsO + MetS	↓ PASI (reported anecdotally)	↓ BMI	Discusses immunometabolic rationale for GLP-1RAs use
Nicolau et al. (53)	2023	Prospective real-world	Liraglutide	20 obese PsO	↓ PASI, CRP, VAS pain	↓ BMI	Regression showed PASI improvement independent of weight loss
Nicolau et al. (54)	2025	Prospective open−label cohort, 6−month duration	Liraglutide (3 mg/day)	48 PsO + obese	↓ PASI + BDI	↓ BMI + waist circumference + pre−peritoneal fat thickness	Improvement in PsO and depressive symptoms occurred. Multiple regression showed dermatological improvement was independent of weight loss, indicating immunomodulatory effects beyond metabolic control
Nicolau et al. (61)	2025	Prospective open−label cohort (3 months)	Liraglutide (3 mg/day)	10 PsA + obese	Improvement in MDA	↓ BMI + waist circumference + glycemia + lipid levels + CRP	Small sample; observed benefit likely mediated *via* inflammation reduction
Petković− Dabić et al. (45)	2025	Open−label randomized clinical trial	Semaglutide (1 mg/week)	31 PsO + T2DM + PsO (n=15 semaglutide; n=16 control)	↓PASI; ↓ in serum IL−6 and CRP	↓BMI + LDL+ glycemia	Systemic anti−inflammatory and metabolic benefits
Sontam et al. (60)	2025	Retrospective cohort via TriNetX (2014–2024)	Any GLP−1RA	PsO (GLP-1RAs users *vs* matched non−users)	↓ incidence of 10 year composite MACE	Not directly assessed	Lower incidence of MACE in users *vs* controls

AMPK, AMP-activated protein kinase; BDI, Beck Depression Index; BMI, Body mass index; CRP, C-reactive protein; GLP-1, Glucagon-like peptide-1; GLP-1RA, Glucagon-like peptide-1 receptor agonist; HbA1c, Haemoglobin A1c; iNKT cells, Invariant natural killer T cells; MACE, Major adverse cardiovascular events; MetS, Metabolic syndrome; MDA, Minimal Disease Activity; N/A, Not Available; PASI, Psoriasis Area and Severity Index; PsA, Psoriatic arthritis; PsO, Psoriasis; T2DM, Type 2 diabetes mellitus; VAS, Visual analogue scale; LDL, Low-density lipoprotein.

↓ reduction; ↑ increase.

## 2 Results

Several studies have explored the therapeutic potential of GLP-1RA in patients with PsO, especially those with comorbid T2DM or MetS (39–45).

To provide context, GLP-1RAs comprise a heterogeneous class of incretin-based agents approved for the treatment of T2DM and, more recently, obesity. The main agents currently approved by the EMA and/or FDA include exenatide (2005/2005), liraglutide (2009/2010), lixisenatide (2013/2016), dulaglutide (2014/2014), semaglutide (2017/2017; oral formulation 2019), and tirzepatide (2022)—the latter being a dual GIP/GLP-1 agonist (**Table 2**) (46).

**Table 2:** Overview of GLP-1 receptor agonists (GLP-1RAs) approved by the EMA and/or FDA, with year of authorization and main therapeutic indications.

Molecule	Type	EMA approval	FDA approval	Main indications
Exenatide	Short-acting GLP-1RA	2006	2005	T2DM
Liraglutide	Long-acting GLP-1RA	2009	2010	T2DM, Obesity (2014)
Lixisenatide	Short-acting GLP-1RA	2013	2016	T2DM
Dulaglutide	Long-acting GLP-1RA	2014	2014	T2DM
Semaglutide	Long-acting GLP-1RA	2018	2017	T2DM, Obesity (2021)
Tirzepatide	Dual GIP/GLP-1 agonist	2023	2022	T2DM, Obesity

EMA, European Medicines Agency; FDA, U.S. Food and Drug Administration; GIP, glucose-dependent insulinotropic polypeptide; GLP-1RA, glucagon-like peptide-1 receptor agonist; T2DM, type 2 diabetes mellitus.

Early studies suggested a mechanistic link between GLP-1 signaling and adipose tissue inflammation, a process increasingly recognized as relevant to the pathogenesis of PsO (47). Adipose-derived inflammation contributes to systemic low-grade inflammation through the release of cytokines such as TNF-α, IL-6, and IL17, which can exacerbate keratinocyte activation and immune dysregulation in psoriatic skin; these cytokines are produced primarily by adipocytes, tissue-resident macrophages, and infiltrating immune cells (e.g., Th17 cells and other T lymphocytes) within the adipose tissue, which can exacerbate keratinocyte activation and immune dysregulation in psoriatic skin (48). GLP-1 receptor activation has been shown to counteract these mechanisms by reducing macrophage infiltration in adipose tissue, suppressing NF-κB–mediated cytokine release, and promoting an anti-inflammatory immune profile (49). These findings suggest that part of the clinical benefit of GLP-1RAs in PsO may derive not only from metabolic improvements but also from direct modulation of adipose-driven inflammatory pathways. These findings were reported in clinical and experimental studies investigating the possible influence of GLP-1RAs on both skin inflammation and metabolic markers (50, 51).

### 2.1 Clinical studies on liraglutide

All selected studies were on PsO patients In 2011, Hogan et al. (39) first reported improvements in psoriasis area and severity index (PASI) scores in two patients with PsO and T2DM, demonstrating that liraglutide reduced cytokine secretion and modulated invariant natural killer T cells (iNKT). Similarly, following studies observed significant clinical improvement in PsO patients with obesity and T2DM, linking improvements to both weight loss and direct immunomodulation (40). In a subsequent prospective cohort, liraglutide not only improved metabolic parameters but also demonstrated immunomodulatory effects, with a significant reduction in circulating T-cell populations, particularly γδ T cells, which are known to play a key role in PsO pathogenesis by producing IL17 and IL22 (26). The decrease in these pro-inflammatory lymphocytes paralleled clinical improvement in skin lesions, suggesting that GLP-1 receptor activation may directly modulate immune cell function in addition to improving metabolic inflammation (26).

Successive case reports confirmed the beneficial effects of liraglutide in obese PsO patients without T2DM (52), and a subsequent trial demonstrated marked improvements in PsO symptoms within a few days of treatment, regardless of significant weight loss (50), a result which was later confirmed in other studies (53, 54).

Subsequent studies confirmed that liraglutide significantly improved PASI scores and histologic features of PsO, mainly epidermal thickness, inflammatory cell infiltration, and keratinocyte hyperproliferation (42, 55). Together with the expected decrease of body mass index (BMI), waist circumference, and glycemic markers, a number of studies confirmed a reduction of inflammatory markers such as IL17 and TNF-α, reinforcing the immunomodulatory action of GLP-1RAs, both in murine (56) and human models (55).

### 2.2 Clinical studies on semaglutide

All selected studies were on PsO patients. Semaglutide, another GLP-1RA, appeared to be promising for treating PsO. Costanzo et al. (57) first reported dramatic improvement in PsO symptoms in a 73-year-old patient who had previously been unresponsive to biologic therapy. More recent studies, also found that semaglutide reduced visceral adiposity and associated epicardial inflammation on imaging study, suggesting that GLP-1RAs may address both skin and fat-related inflammation in PsO patients with obesity and T2DM (58). Additionally, a recent open-label randomized trial showed that semaglutide significantly reduced serum IL-6 and CRP levels in PsO T2DM patients, compared to controls not on semaglutide therapy, reinforcing the anti-inflammatory effects of GLP-1RAs (45).

### 2.3 Long-term and real-world evidence

Long-term evidence from selected studies, highlights the sustained clinical benefits of liraglutide and semaglutide (45, 53).

A number of studies provided compelling evidence that liraglutide significantly improved PASI scores, CRP levels, and VAS pain in obese patients with PsO, regardless of weight loss, highlighting the direct immunomodulatory role of GLP-1Ras (50, 53, 54). These findings are supported by a previous study, reporting a reduction in PASI, despite not being statistically significant, together with substantial improvement of BMI and cholesterol levels in an open-label trial of liraglutide in obese PsO patients (59). Notably, the paper reported a clear dose–response relationship, further suggesting a pharmacological effect of GLP-1RAs on inflammatory pathways in PsO including metabolic benefits (59).

Finally, in a large scale retrospective cohort study by Sontam et al. (60), the use of GLP-1RA was associated with a lower incidence of major adverse cardiovascular events (MACE) in PsO patients compared to matching controls, further emphasizing the systemic benefits of GLP-1RAs in addition to skin improvement. Notably, this study did not include data specifically addressing early PsO.

### 2.4 Comment on psoriatic arthritis study

While the majority of studies focus on PsO and its association with T2DM or MetS, there is only one small study specifically investigating PsA. This study by Nicolau et al. (61), evaluated the effects of liraglutide (3 mg/day) for 3 months in obese PsA patients. Although disease duration was not reported, the participants were not characterized as early PsA. The study found that liraglutide resulted in improvement in minimal disease activity scores, decreasing BMI, waist circumference, glycemia, lipid levels, and inflammatory markers. Despite the small sample size (only 10 patients), the observed benefits suggest that GLP-1RAs could have a therapeutic role in PsA through inflammation reduction, similar to what had been observed in PsO (61).

This highlights a gap in the research, as PsA has been less extensively studied with GLP-1RAs, and further studies are needed to confirm whether GLP-1RAs provide similar immunomodulatory and metabolic benefits in PsA as they do in PsO. The current evidence indicates promising potential, however a broader validation is required, specifically in the context of PsA.

## 3 Discussion

### 3.1 GLP-1RAs in psoriasis

The exploration of GLP-1RAs in the treatment of PsO and PsA has emerged as a promising area of research. These agents, known for their dual impact on both immune modulation and metabolic improvement, offer a unique therapeutic potential in managing immune-mediated inflammatory diseases (47). While the majority of current studies focus on PsO, the potential benefits of GLP-1RAs in PsA are not adequately studied, raising important questions about their broader applicability (61).

### 3.2 GLP-1RAs in psoriatic arthritis

The therapeutic landscape of PsA is currently dominated by targeted agents that inhibit key inflammatory pathways, including TNF inhibitors (e.g., adalimumab, etanercept), IL17 inhibitors (e.g., secukinumab, ixekizumab), IL-23 inhibitors (e.g., guselkumab, risankizumab), and Janus kinase (JAK) inhibitors such as upadacitinib and tofacitinib (11, 62). While these therapies effectively modulate the inflammatory cascade underlying PsA, accumulating evidence suggests that metabolic factors—particularly BMI—may significantly influence clinical response.

Several studies have reported an inverse relationship between BMI and therapeutic efficacy, indicating that obesity may attenuate clinical responses to biologic disease modifying anti-rheumatic drugs (bDMARDs). This observation has raised concerns regarding suboptimal drug exposure, altered pharmacokinetics, and heightened inflammatory burden in obese patients, highlighting the potential need for dose optimization or alternative treatment strategies in this population (63, 64).

The impact of BMI on treatment response is well illustrated by the AQUILA study, which examined real-world outcomes in PsA patients treated with (65).In this cohort, obese individuals exhibited higher circulating levels of IL-17, supporting the concept that IL-17–driven inflammation may be particularly prominent in this subgroup and may contribute to increased disease activity and reduced therapeutic responsiveness (66). These findings reinforce the notion that metabolic dysfunction, and obesity in particular, is not merely a comorbidity but an active modifier of PsA pathophysiology and treatment outcomes.

However, the relationship between BMI and response to biologic therapies is not uniformly consistent across studies. Some real-world cohorts and long-term extension trials have reported comparable efficacy of biologic agents across BMI categories, especially when weight-based dosing regimens or dose optimization strategies were applied. These divergent findings likely reflect heterogeneity in study design, mechanisms of action of the drugs evaluated, duration of follow-up, and the degree of metabolic phenotyping across populations.

Taken together, these data suggest that obesity does not universally impair biologic efficacy but remains consistently associated with a higher inflammatory burden and increased comorbidity load. Even in settings where clinical response to biologics is preserved, the presence of obesity provides a strong rationale for adjunctive metabolic interventions. In this context, therapies such as GLP-1RAs may represent a complementary strategy aimed at modulating both metabolic dysfunction and inflammation, thereby optimizing disease control and improving long-term outcomes in PsA.

### 3.3 Metabolic modulation and immunomodulatory effects

The close relationship between BMI, metabolic dysfunction, and immune activation has important implications for the potential use of GLP-1Ras in obese patients with PsA. In PsO, clinical studies evaluating GLP-1RAs such as liraglutide and semaglutide have reported significant improvements in PASI scores and histopathological skin features, effects that appear to be at least partially independent of weight loss (50, 53, 54). These observations suggest that GLP-1RAs may exert direct immunomodulatory actions beyond their metabolic effects.

Given the prominent role of IL-17–driven inflammation in obese PsA patients, GLP-1RAs may also enhance the efficacy of biologic agents targeting these pathways. By concurrently modulating metabolic dysfunction and inflammatory signaling, GLP-1RAs could potentially reduce the required doses of biologics or other immunosuppressive therapies, thereby minimizing treatment-related toxicity while improving overall disease control. This dual action positions GLP-1RAs as attractive adjunctive agents capable of optimizing therapeutic strategies across different stages of PsA.

The relevance of metabolic modulation for treatment response is further supported by evidence showing that metabolic status significantly influences the effectiveness of systemic therapies in PsO. In a seminal study, Gisondi et al. demonstrated that intentional weight loss significantly improved the response of obese PsO patients to low-dose cyclosporine (67) This finding underscores the broader concept that improving metabolic health can enhance responsiveness to immunosuppressive treatments and provides a rationale for combining metabolic interventions with systemic therapies, including GLP-1RAs.

Collectively, these data highlight the importance of integrating metabolic health into early therapeutic strategies for PsA, particularly in individuals at high risk of disease progression or in those presenting with subclinical musculoskeletal involvement. By improving metabolic function, GLP-1RAs may not only enhance disease control but also optimize responses to concomitant therapies during the critical early phases of PsA. Such an integrated approach supports a more holistic and potentially preventive strategy for addressing both the immune and metabolic components of the disease.

Importantly, while weight loss represents a major contributor to the clinical benefits of GLP-1RAs, accumulating evidence indicates that these agents also exert non-weight-dependent effects. Beyond absolute reductions in body weight, improvements in insulin sensitivity appear to be a key mechanistic link between metabolic interventions and attenuation of systemic inflammation. Enhanced insulin signaling following weight loss has been associated with decreased circulating levels of pro-inflammatory cytokines, including IL-6, TNF-α, and C-reactive protein, as well as improvements in endothelial and adipose tissue function (68).

In inflammatory arthritis, including PsA, improved insulin sensitivity has been independently associated with reduced disease activity and inflammatory burden, even after adjustment for changes in BMI (69). These observations support the hypothesis that GLP-1RAs may exert anti-inflammatory effects partly through the restoration of insulin responsiveness, rather than through weight reduction alone.

Consistent with this concept, GLP-1RAs have been shown to directly modulate immune responses by reducing the secretion of key pro-inflammatory cytokines such as IL-6 and TNF-α, which are central drivers of inflammation in both PsO and PsA (70, 71). These immunomodulatory properties may enhance the effectiveness of conventional biologic therapies and could potentially support disease control in early PsA, although this possibility still requires confirmation in dedicated clinical studies, a phase in which immune dysregulation is still evolving. GLP-1RAs appear to offer a potential dual therapeutic advantage, simultaneously targeting metabolic dysfunction and immune-mediated inflammation, thereby representing a potentially promising strategy in the management of early-stage PsA early PsA, although robust clinical evidence in this setting is currently lacking. Taken together, these findings should be interpreted cautiously, as most available data derive from small studies or indirect evidence and may not be directly generalizable to early PsA populations.

### 3.4 Early PsA and future research directions

Despite the promising metabolic and immunomodulatory effects observed in PsO, the role of GLP-1RAs in PsA, particularly in its early stages, remains largely unexplored. Increasing attention has been directed toward the concept of a therapeutic “window of opportunity,” which frames early PsA as a transitional phase characterized by musculoskeletal symptoms such as arthralgia, dactylitis, and subclinical enthesitis in patients with PsO, often preceding clinically evident arthritis (12). Intervening during this stage by combining GLP-1RAs with established therapeutic strategies could theoretically provide a dual benefit by simultaneously modulating immune activation and correcting metabolic dysfunction, with the potential to prevent or delay disease progression; however, this hypothesis remains to be confirmed in prospective studies specifically targeting early PsA populations.However, emerging data also underscore the complexity of this therapeutic hypothesis. A recent study by Olbrich et al. (72), reported that GLP-1RA treatment in PsO patients receiving systemic therapy did not significantly alter the subsequent risk of developing PsA. Several factors may account for these findings. First, PsO patients already treated with biologic agents—such as TNF or IL-17 inhibitors—may experience sufficient immune suppression to limit any additional protective effect of GLP-1RAs on PsA development. Second, although PsO and PsA share overlapping inflammatory pathways, they are characterized by partially distinct immune mechanisms; consequently, the immunomodulatory effects of GLP-1RAs on cutaneous inflammation may not directly translate to the pathways driving joint and entheseal disease.

These considerations highlight the need for future studies specifically designed to evaluate GLP-1RAs in patients at high risk of PsA, particularly those with subclinical inflammation or early musculoskeletal symptoms. Targeting this population would allow a more accurate assessment of whether metabolic modulation can meaningfully influence immune dysregulation at a stage when disease processes may still be reversible. Importantly, the current evidence supporting this approach is largely indirect and derived from studies conducted in patients with established PsO and metabolic comorbidities, rather than in well-characterized early PsA cohorts.An additional and largely underexplored aspect concerns the safety of combining GLP-1RAs with bDMARDs. To date, available clinical experience —primarily derived from populations with T2DM or obesity receiving concomitant immunomodulatory therapies— has not revealed major safety signals related to increased infection risk or immune suppression. Importantly, GLP-1RAs do not exert direct immunosuppressive effects but instead modulate inflammatory pathways indirectly through metabolic and cellular mechanisms, supporting a favourable safety profile when used alongside bDMARDs. Ongoing studies, including TOGETHER-PsA and TOGETHER AMPLIFY-PsA, are expected to generate critical real-world data on tolerability, metabolic–immune interactions, and long-term safety of this combined therapeutic approach. Nevertheless, robust clinical evidence in PsA remains limited, underscoring the need for dedicated randomized controlled trials to clarify the effects of GLP-1RAs on joint inflammation, disease progression, and long-term clinical outcomes. Future research should also address optimal dosing, timing of intervention, and potential synergistic or antagonistic interactions with existing immunosuppressive therapies—areas that remain largely unexplored but could have major implications for clinical practice. Beyond disease activity, metabolic health may also influence symptom burden and quality of life in PsA. Emerging evidence suggests that MetS contributes to pain catastrophizing, with obesity acting as a key amplifier of pain perception in inflammatory diseases such as PsA (73). Visceral adiposity is strongly associated with systemic inflammation, and its reduction may yield both metabolic and anti-inflammatory benefits (74). Moreover, improvements in insulin sensitivity—achievable through GLP-1RAs—may mitigate pain-related symptoms, as insulin resistance has been implicated in chronic inflammation and heightened pain perception (75). By addressing both immune-mediated inflammation and metabolic drivers of pain, GLP-1RAs may therefore offer a dual therapeutic advantage extending beyond disease control to overall patient well-being.

In addition to psoriatic disease, GLP-1RAs are currently under investigation in other immune-mediated inflammatory diseases (IMIDs), such as inflammatory bowel disease, RA, and systemic lupus erythematosus (70, 71, 76). Recent reviews have highlighted potential immunomodulatory effects across diverse autoimmune contexts, suggesting that the observed anti-inflammatory properties may extend beyond metabolic improvement (77). This raises the intriguing possibility of a class effect, where GLP-1RAs could represent a novel therapeutic approach across multiple IMIDs, given the overlap in immunometabolic mechanisms common to these diseases. As such, further studies should explore whether the beneficial effects observed in PsO and early PsA could be replicated in other autoimmune disorders, providing broader therapeutic benefits across this category of diseases.

Several clinical trials registered on ClinicalTrials.gov support the growing scientific interest in the repurposing of GLP-1RAs for psoriatic disease. Notably, completed and ongoing studies such as NCT06475586, NCT06937060, NCT06588296, and NCT06864026 are investigating semaglutide in patients with PsO and comorbid metabolic conditions, or tirzepatide (in PsA settings), assessing dermatologic outcomes and inflammatory or metabolic markers (78, 79). NCT06588296 (TOGETHER-PsA) (80) is a Phase 3b, randomized, multicentre, open-label study examining the efficacy and safety of ixekizumab compared to ixekizumab concomitantly administered with tirzepatide in overweight or obese adult patients with active PsA and at least one weight-related comorbidity. Its design includes parallel arms and aims to assess whether the addition of tirzepatide may be beneficial for both joint outcomes and weight/metabolic parameters in this particular PsA population.

NCT06864026 (TOGETHER AMPLIFY-PsA) (81) is a Phase 4, prospective, open−label, single−arm “real -world” study in which tirzepatide is added to ongoing ixekizumab therapy in patients with active PsA who are overweight or obese (BMI ≥ 30 kg/m², or 27–<30 with at least one weight−related comorbidity). The main aim is to assess the effectiveness (in a clinical practice setting) of this combined approach up to 12 months, focusing on articular disease control as well as metabolic change (e.g. weight loss) in a “real-world” PsA population.

Although direct evidence for GLP-1RA use in PsA remains limited, the inclusion of PsA populations in these tirzepatide trials represents a conceptual shift toward testing immunometabolic interventions in inflammatory arthritis rather than restricting their evaluation to skin disease alone. The discontinued NCT02472717 trial investigating liraglutide in PsA further illustrates both the pioneering nature and the challenges inherent in this research field (82).

Importantly, none of the currently available trials specifically target the early PsA window, which is increasingly recognized as a critical period for disease modification. This gap highlights a key unmet need identified by the present review. Future studies should prioritize early PsA populations, ideally using stratified designs that incorporate metabolic profiling, to fully elucidate the dual immunomodulatory and metabolic potential of GLP-1RAs in altering disease trajectory. Finally, it should be acknowledged that a universally accepted definition of early PsA is still lacking; while the EULAR “Points to Consider” provide valuable guidance, the development of standardized criteria will be essential to advance research in this area.

## 4 Limitations

This review displays some limitations which should be acknowledged. It is a narrative rather than a systematic review. This approach was chosen deliberately, given the current scarcity of randomized controlled trials and the limited number of clinical studies specifically addressing GLP-1RAs in PsA. Evidence on PsA remains limited, with only one open-label clinical study available to date. Most published studies are small, involve patients affected by obesity and/or T2DM, and may therefore not be fully generalizable to the broader PsO/PsA population. Furthermore, despite GLP-1RAs have consistently contributed to the improvement of PsO severity and systemic inflammation, disentangling metabolic-driven benefits from direct immunomodulatory effects is still a challenge.

In spite of these limitations, the available data provide a strong rationale for further investigation of GLP-1RAs in psoriatic disease. Future large-scale, well-designed clinical trials will be essential to confirm their therapeutic potential, clarify the contribution of metabolic versus immunological effects, and define their role within the treatment algorithm of PsO and PsA.

## 5 Future directions

The potential of GLP-1RAs as dual-action therapies, targeting both metabolic dysfunction and immune dysregulation, makes them particularly compelling candidates for early PsA, where intervention may alter disease trajectory. Given the recognized “window of opportunity” in early PsA, future research should prioritize trials in patients with subclinical joint involvement, arthralgia, or early enthesitis, especially those with coexisting obesity or MetS. Key areas of investigation include optimal timing of initiation, identification of responsive subgroups of patients, and evaluation of synergistic effects when combining GLP-1RAs with established biologics. Long-term studies are also needed to assess whether GLP-1RAs can prevent progression from PsO to PsA, improve musculoskeletal outcomes, and reduce systemic comorbidities such as cardiovascular disease. Clarifying these aspects will be essential to define the clinical utility of GLP-1RAs in PsA and potentially across other immune-mediated inflammatory diseases.

## 6 Conclusion

The encouraging results observed in PsO suggest that GLP-1RAs may have broader applications in managing immune-mediated diseases such as PsA; however, their role in this context remains to be clearly defined through dedicated clinical studies. This approach reflects the success seen with SGLT2 inhibitors, which have demonstrated significant cardioprotective and anti-inflammatory effects in conditions such as SLE, leading to their integration into clinical practice for autoimmune diseases (83). The intersection of metabolic dysfunction and psoriatic disease represents a promising frontier for therapeutic innovation. To fully achieve the potential benefits of GLP-1RAs in PsA, prospective studies are needed, particularly focusing on early PsA patients to evaluate their ability to halt or even reverse musculoskeletal progression. Furthermore, combination therapy trials exploring the synergistic effects of GLP-1RAs with biologics could uncover new approaches for treatment, enhancing the management both metabolic and immunologic aspects in PsA.
