# Supplementary material for: Glucagon-like peptide-1 receptor agonists in psoriasis and psoriatic arthritis: emerging evidence and future research opportunities
Source: Front Immunol. 2026 Apr 28;17:1744308. doi: 10.3389/fimmu.2026.1744308 (PMC13161150; doi:10.3389/fimmu.2026.1744308)
Supplement: Supplementary file 1 [file Table1.docx]

Supplementary Material

# Search strategy (conducted on 4^th^ August 2025, with a specific limitation to studies published in English) on Pubmed, Embase, Cochrane Library

# ("glucagon like peptide 1 receptor agonist"[Title/Abstract] OR "glucagon-like peptide 1 receptor agonist"[Title/Abstract] OR "glucagon like peptide-1 receptor agonist"[Title/Abstract] OR"glucagon-like peptide-1 receptor agonist"[Title/Abstract] OR "GLP1 receptor agonist"[Title/Abstract] OR "GLP-1 receptor agonist"[Title/Abstract] OR "GLP 1 receptor agonist"[Title/Abstract] OR "glucagon like peptide 1 analog"[Title/Abstract] OR "GLP1 analog"[Title/Abstract] OR "GLP-1 analog"[Title/Abstract] OR "liraglutide"[Title/Abstract] OR "exenatide"[Title/Abstract] OR "semaglutide"[Title/Abstract] OR "dulaglutide"[Title/Abstract] OR "lixisenatide"[Title/Abstract] OR "albiglutide"[Title/Abstract])AND ("psoriatic arthritis"[Title/Abstract] OR "psoriasis"[Title/Abstract] OR "axial spondyloarthritis"[Title/Abstract] OR "ankylosing spondylitis"[Title/Abstract])

## Supplementary Figures


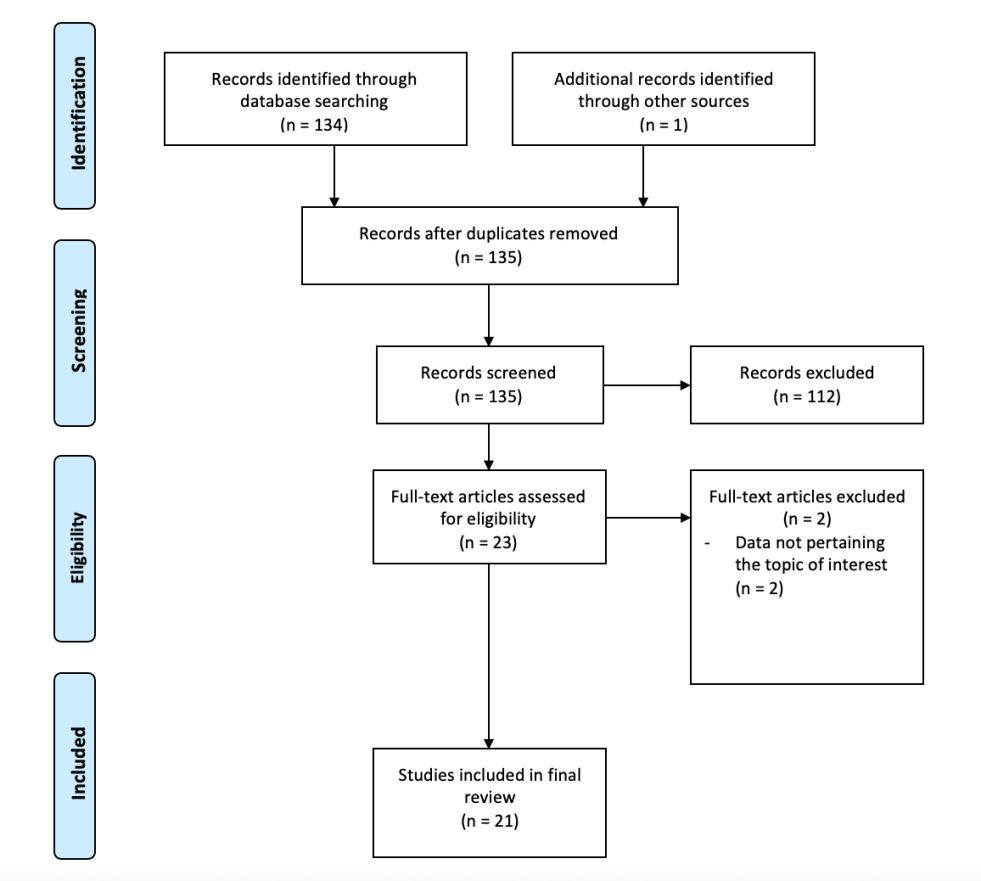


**Supplementary Figure 1.** Selection process: flow diagram describing the inclusion decision of papers under the scope of this review.
